# A set of SSR markers to characterize genetic diversity in all *Viburnum* species

**DOI:** 10.1038/s41598-023-31878-0

**Published:** 2023-04-01

**Authors:** Trinity P. Hamm, Marcin Nowicki, Sarah L. Boggess, Thomas G. Ranney, Robert N. Trigiano

**Affiliations:** 1 Department of Entomology and Plant Pathology, University of Tennessee, Knoxville, TN 37996 USA; 2 Mountain Crop Improvement Lab, Department of Horticultural Science, Mountain Horticultural Crops Research and Extension Center, North Carolina State University, 455 Research Drive, Mills River, NC 28759-3423 USA

**Keywords:** Genetic markers, Plant genetics

## Abstract

About 160 species are classified within the *Viburnum* genus and many of these are cultivated for horticultural purposes. The vast dispersal of *Viburnum* makes the genus a useful model for studying evolutionary history and inferring how species expanded into their current distributions. Simple sequence repeat (SSR) markers were previously developed for five *Viburnum* species that were classified within the four major clades (*Laminotinus*, *Crenotinus*, *Valvatotinus*, and *Porphyrotinus*). The ability of some of these markers to cross-amplify in *Viburnum* species has been scantly evaluated, but there has not been any genus-wide assessment for the markers. We evaluated a collection of 49 SSR markers for the ability to cross-amplify in 224 samples, including 46 *Viburnum* species, representing all 16 subclades, and five additional species in the Viburnaceae and Caprifoliaceae. A subset of 14 potentially comprehensive markers for *Viburnum* species was identified and evaluated for the ability to detect polymorphisms in species outside of their respective clades. The 49 markers had overall amplification success in 52% of the samples, including a 60% success rate within the *Viburnum* genus and 14% in other genera. The comprehensive marker set amplified alleles in 74% of all samples tested, including 85% of *Viburnum* samples and 19% of outgroup samples. To the best of our knowledge, this is the first comprehensive set of markers able to characterize species across an entire genus. This set of markers can be used to assess the genetic diversity and population structure of most *Viburnum* species and closely allied species.

## Introduction

*Viburnum* L. was formerly classified within the Caprifoliaceae (honeysuckle family) alongside *Lonicera* L. and *Weigela* Thunb. *Viburnum* is a large genus comprised of approximately 160 species^1^, now classified in the Viburnaceae (formerly Adoxaceae)^2,3^ with *Adoxa* L., *Sambucus* L., and a few other genera. Species within *Viburnum* are native to temperate and subtropical regions of the Northern Hemisphere, and the range extends into the mountainous regions of Southeastern Asia and South America. The somatic chromosome numbers of *Viburnum* species range from 18 to 72^4,5^ and in ploidy level from diploid to octaploid. Not all species have been equally studied, but most of the species are 2*n* = 2*x* = 18^4–6^. Studied *Viburnum* genome sizes are classified as small to intermediate when compared to plants overall and have 2C values ranging from 4.29 to 24.23 Gbp^7–11^. However, the genome sizes are larger than some other woody genera, such as *Cornus* with 2C values ranging from 1.89 to 6.66 Gbp^7,12–14^ and *Pyrus* ranging from 1.13 to 1.27 Gbp^10,15,16^.

The genus *Viburnum* includes many species of shrubs to small trees with year-round ornamental qualities, which are grown for their fragrant flowers and attractive foliage. There are 70 species and interspecific hybrids in cultivation^17^ that generated $23.2 million in wholesale and retail sales in 2019^18^. This well-sampled, geographically widespread, and abundant genus also provides a model for addressing evolution, biogeography, phylogenetics, and ecology-related questions, and has been the topic of many investigations^1,19–22^.

Much progress has been made in the phylogenetic classification of this genus^6,21,23–26^, but knowledge gaps persist in the taxonomy that if resolved, would provide better insight into the evolution, biogeography, and phylogenetics of the genus. DNA-barcoding was implemented but had a low success rate due to low sequence variability^25^. Restriction-site-associated DNA sequencing (RADseq) was applied to the *V. nudum* species complex in North America and was successful in identifying three independent lineages in support of three separate species^27^. However, this next generation sequencing (NGS) approach may not be feasible for every species in this genus because of the relatively large costs associated with NGS in addition to the large genome sizes and ploidy levels of some *Viburnum* spp.

**Figure 1:**
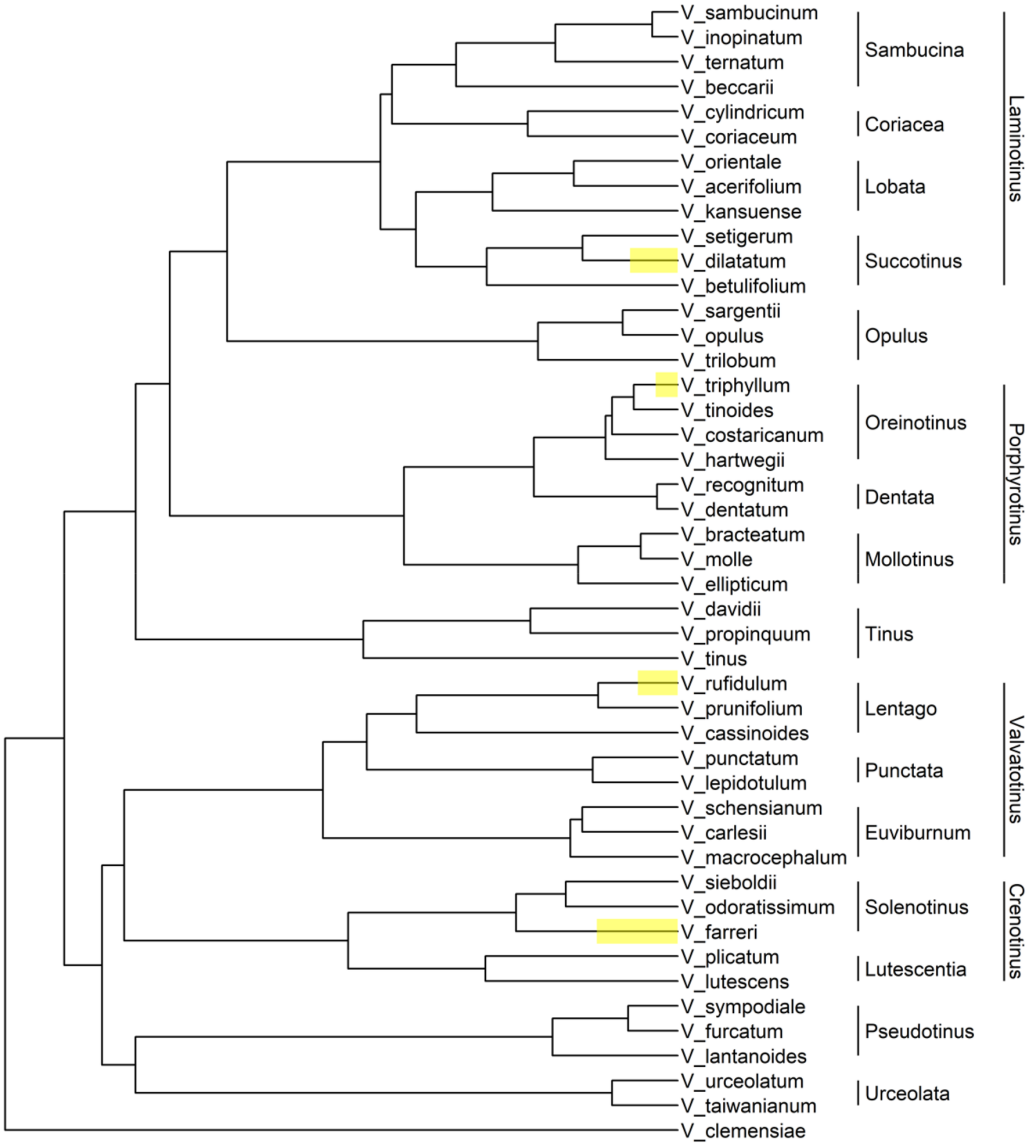
Phylogenetic tree of *Viburnum* species included in this study. This tree is a trimmed version of the one published in Landis et al.^1^ based on RAD-seq data. Highlighted nodes are species in which simple sequence repeat (SSR) markers were developed. Subclades are horizontal and major clades are vertical.

Four sets of microsatellite markers, also known as simple sequence repeat (SSR) markers, were developed from species within the four major clades of *Viburnum*^22^ including *Laminotinus* (*Succotinus* subclade)^28^, *Valvatotinus* (*Lentago* subclade)^29^, *Porphyrotinus* (*Oreinotinus* subclade)^30^, and *Crenotinus* (*Solenotinus* subclade)^31^ (Fig. 1). SSRs consist of short nucleotide motifs that are tandemly repeated. The nucleotide motifs can be between one and five base pairs (bp) long and are ubiquitous throughout the genome^32^. These regions of the genome can accumulate mutations faster than others predominantly due to polymerase slippage during DNA replication and are flanked by relatively conserved genetic sequences, thus allowing for the design of PCR primers to consistently amplify a given locus^33,34^. The resulting amplicons can then be sized or sequenced to determine the alleles in an individual. The co-dominant nature, hypervariability, reproducibility, and PCR applicability make these genetic markers a popular option for population analyses^35^, species and cultivar delimitation^36^, and breeding^37^. Furthermore, SSR markers often transfer to closely related species and genera, and only require small amounts of low-quality DNA for successful amplification. A cost analysis was performed in 2020 on SSR markers used in conjunction with the QIAxcel capillary electrophoresis system and determined the cost per sample per locus to be less than $12^38^, making this a very affordable option. The combination of high polymorphism, low cost, and low-quality DNA requirements make SSRs an optimal tool for the vast *Viburnum* genus.

Using SSR markers developed in other species to study related species is a common practice and has been demonstrated in various ornamental taxa such as *Cornus* (dogwood)^39^ and *Cercis* (redbud)^40^ as well as food crops such as *Glycine* (soybean)^33^, *Prunus* (stonefruit)^41^, and *Foeniculum* (fennel)^42^. A meta-analysis was completed on studies reporting cross-species amplification success in plants, fungi, and animals, and determined for eudicots overall. For eudicots the percentage of markers that were able to cross-amplify in species within genera was almost 80% and almost 60% among genera, whereas the percentage of markers that were actually polymorphic was almost 60% within genera and 10% among genera^43^. Some of the *Viburnum* markers were demonstrated to cross-amplify in their initial development publications, and a preliminary cross-amplification analysis was performed as part of Dean’s dissertation^44^. Additionally, the markers developed from *V. dilatatum* were used to amplify alleles in *V. opulus, V. trilobum,* and *V. sargentii*^45^, but no large-scale analysis has been published with any of the four sets.

The development of unique marker sets for each of the approximately 160 *Viburnum* species^1^ would be cost- and time-prohibitive. Therefore, the overarching goal of the study was to develop a set of comprehensive markers that could be applied to studies of genetic diversity, population genetics, and potentially phylogenetics of all the species within *Viburnum* as well as some closely allied genera in the Viburnaceae and Caprifoliaceae. We hypothesized this comprehensive marker set could be identified from the previously published markers. To test this the following aims were completed: 1) evaluate the cross-amplification of the 49 previously published markers in 46 *Viburnum* species and five species in the Viburnaceae and Caprifoliaceae; 2) compile a set of comprehensive markers for the genus, and 3) demonstrate the ability of the comprehensive marker set to detect polymorphisms in species outside of the subclades from which they were developed.

## Materials and methods

### Plant materials and gDNA extraction

Leaf samples were donated by arboreta, herbaria, and public universities across the U.S. except for the few species found locally in East Tennessee, U.S. (Supplementary Table 1). The use of samples in the present study complied with international, national and/or institutional guidelines. Local samples were collected and identified by Robert Trigiano and William Klingeman from public areas, which did not require permission to collect (see Supplementary Table 1 for additional information). The goal was to obtain three independent samples for each species and three species from each of the 16 subclades, following the classification scheme proposed in Clement et al.^22^ (i.e., *Lentago*, *Punctata, Euviburnum, Pseudotinus, Urceolata, Solenotinus, Lutescentia, Tinus, Sambucina, Coriacea, Succotinus, Lobata, Opulus, Mollotinus, Dentata,* and *Oreinotinus).* For the purposes of this study, these 16 subclades will be referred to as subclades and *Valvatotinus*, *Crenotinus*, *Porphyrotinus*, and *Laminotinus* will be referred to as major clades, despite there being higher classifications than these four. For additional information about the formal phylogenetic definitions, see Clement et al.^22^. Due to limited sampling of some subclades, it was not possible to meet the sampling goal for every subclade, but 46 *Viburnum* species and five closely related species in the Viburnaceae and Caprifoliaceae were obtained for a total of 224 samples (Supplementary Table 1). The Viburnaceae and Caprifoliaceae are both classified within Dipsacales, making Caprifoliaceae species ideal candidates for outgroup samples. For visualization of relatedness within *Viburnum*, the phylogenetic tree from Landis et al.^1^ was trimmed with the R packages *ape* (version 5.6–1) and *ggtree* (version 3.2.1) to only include the *Viburnum* species represented in this study (Fig. 1).

Genomic DNA (gDNA) isolations followed the same protocol as described in Hamm et al.^31^. Leaf samples were frozen in liquid nitrogen before homogenization using a Beadmill 24 (Fishers Scientific, Pittsburgh, Pennsylvania, U.S.). All leaf samples from arboreta were air-dried between pieces of newspaper before freezing. gDNA was extracted from leaf samples using the Omega E.Z.N.A. Plant DNA Kit (Omega Bio-tek Inc., Norcross, Georgia, U.S.), following the manufacturer’s protocol except that 2% mass/volume polyvinylpyrrolidone (PVP) (Fisher BioReagents, Waltham, Massachusetts, U.S.) was added to the P1 Buffer and the incubation time at 65 °C was increased from 10 to 30 min. DNA from herbaria samples dating back to 1932 was successfully extracted with this kit. A CTAB protocol^46^ was used with a few herbaria samples because of the paucity of leaf material. The quality of the extracted gDNA was assessed with a NanoDrop Lite Spectrophotometer (Thermo Fisher Scientific, Waltham, Massachusetts, U.S.).

### SSR primers and genotyping conditions

A total of 49 primer pairs from four previous studies that were developed from the four major clades of *Viburnum* were used in this study. Eleven markers were developed from *V. dilatatum* in *Laminotinus*^28^, seven from *V. rufidulum* in *Valvatotinus*^29^, 16 from *V. triphyllum* and *V. pichinchense* in *Porphyrotinus*^30^, and 15 from *V. farreri* in *Crenotinus*^31^ (Supplementary Table 2). All SSR markers were single locus and polymorphic in the species from which they were obtained and included simple-perfect and compound-imperfect motifs. For simplicity, the markers developed from *V. triphyllum* and *V. pichinchense* were renamed Vore01-16 (Vore = *Viburnum Oreinotinus* subclade) in the same order found in the published table (original names in parentheses in Supplementary Table 2)^30^.

PCR was completed with all 49 primer pairs and 224 samples. A reaction volume of 10 µL, consisting of 5 µL of 2X Accustart II PCR SuperMix (Quantabio, Qiagen Beverly, Inc., Beverly, Massachusetts, U.S.), 3 µL autoclaved water, 1 µL of a mixture of 5 µM forward and primers, and 1 µL of 2 ng/µL gDNA was used. A single PCR thermal profile was used with all samples and markers and was as follows: 3 min of initial denaturation at 94 °C, 10 touchdown^47^ cycles (94 °C for 40 s, 60 °C–0.5 °C/cycle for 40 s, and 72 °C for 45 s) and 30 cycles (94 °C for 40 s, 55 °C for 40 s, 72 °C for 45 s), and a final extension at 72 °C for 4 min. This thermal profile was selected as a compromise among all the varying protocols from the initial development publications. PCR products were visualized with capillary electrophoresis (QIAxcel Advanced Electrophoresis System; Qiagen) and analyzed using a 25- to- 500 base pair (bp) DNA size marker (Qiagen) and an internal 15/600 bp alignment marker (Qiagen). Due to the wide variety of species and varying genetic distances among the samples, four positive controls were included in every 96-well PCR plate. The positive control samples were all from freshly collected leaves of species that were used in the original marker development (*V. dentatum, V. dilatatum, V. farreri*, and *V. rufidulum)*. A negative control of sterile water was also included in every plate. Allele sizes were determined using QIAxcel ScreenGel version 1.6.0.10.

### Scoring of amplification and dataset analysis

Only peaks greater than 0.1 Relative Fluorescent Units (RFUs) were considered amplified alleles. For diploid species, only the two strongest peaks were scored. For polyploid species, the number of peaks considered equaled the ploidy level. For example, a total of eight peaks were considered for the octaploid species, *V. bracteatum*. The highest known ploidy in this genus is octaploid, therefore for any species with an unknown ploidy level, a maximum of eight peaks was considered as well. Any amplicon greater than 600 bp was not sized because it was outside the 600 bp maximum of the alignment marker. A reaction was considered within the expected bp range and a successful cross-amplification if the amplicon size was within approximately 50 bp of the expected allele size from the original characterized species. Amplicons outside of the expected range were assumed to be from non-target loci. The 50 bp cutoff was selected because most of the strong peaks with limited noise were within 50 bp of the expected range. If an amplicon was outside of that range, it was recorded as an asterisk in the datasheet. If a reaction resulted in no amplification, the PCR was attempted again for all samples except *L. japonica* and *maackii*. For these outgroup species, if there was no amplification the marker was not repeated, however, if there was amplification with a single sample of these two species, all *L. japonica* and *maackii* samples were repeated. From QIAxcel, the dataset was then imported into Jupyter Notebook for analysis and visualization with custom code (https://github.com/trinityhamm/Viburnum_Cross_Amp/blob/main/Viburnum_Cross_Amp_final.ipynb).

### Validation: comprehensive marker set

Once cross-amplification was completed with all 49 markers, a comprehensive marker set was identified. The 14 markers identified displayed high amplification rates across most subclades. To demonstrate that these markers could characterize species outside of the subclade they were developed in, a subset of the data was analyzed more closely. More than three leaf samples were obtained for *V. carlesii* (n = 14), *V. opulus* (n = 18), *V. plicatum* (n = 15), and *V. tinus* (n = 9), which are all members of subclades where markers were not developed from. The amplification rates and observed heterozygosity were then calculated for each of the 14 comprehensive markers in the four species.

## Results

### Overall dataset

Overall, out of a total of 10,976 potential reactions, 6987 (64%) resulted in ‘any amplification’, and 5711 (52%) produced amplicons within the expected size. ‘Any amplification’ reactions include ones that did not produce any amplicons within 50 bp of the expected range but did produce amplicons outside the range and/or greater than 600 bp. Therefore, 1276 reactions (6987 any amplification—5711 expected size amplifications) only produced amplicons from non-target loci. Twelve percent of the reactions (1361) produced any amplicons outside of the expected bp range. Eight percent or 882 of the reactions produced amplicons greater than 600 bp. The full datasets with allele sizes are broken down by marker set (VD, VF, Vore, and VR; Supplementary Tables 3–6). The maximum number of alleles found per individual was four, despite one species being octaploid. The overall amplification rate for the markers in *Viburnum* was 60% and 14% in samples outside of the *Viburnum* genus.

**Figure 2:**
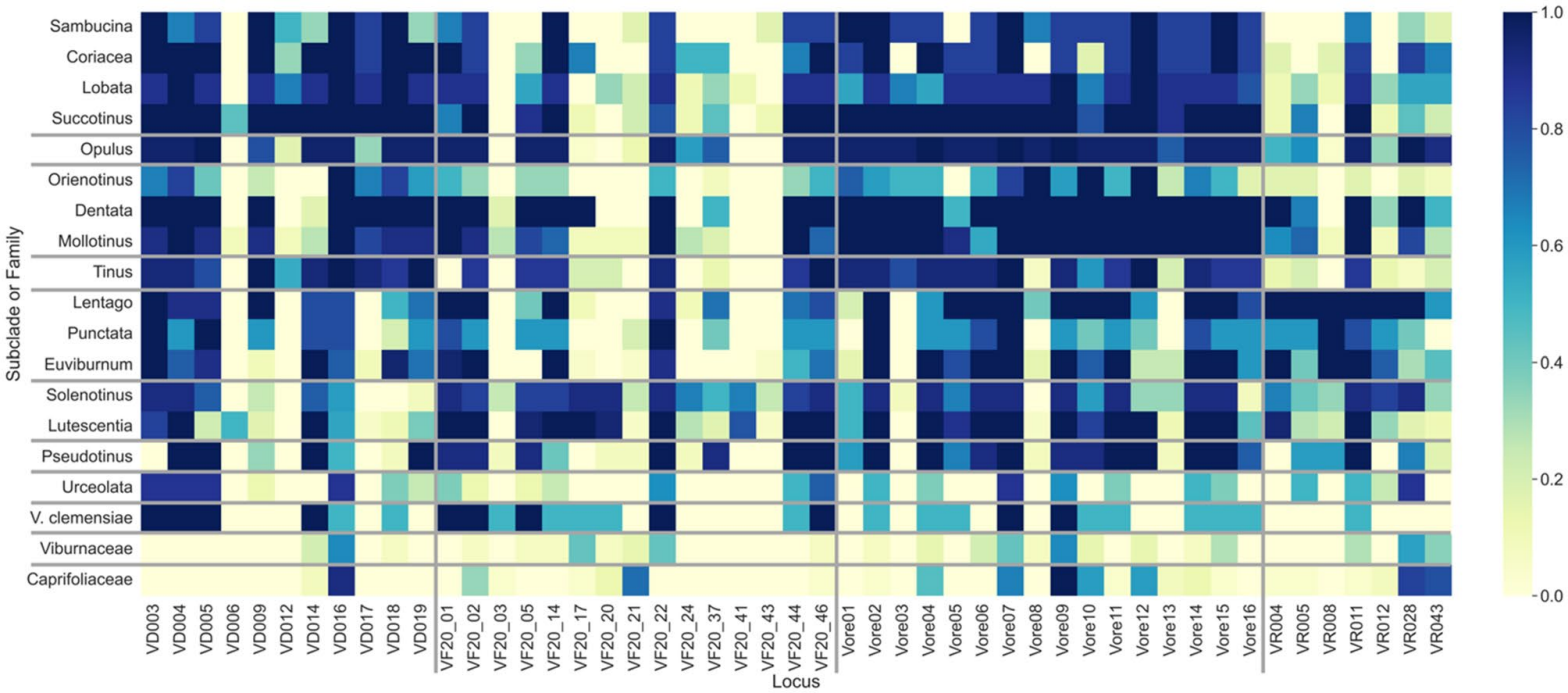
Heatmap of frequency of amplification per subclade or family for each of the 49 markers (loci). Vertical grey lines mark the separation of marker set groups and horizontal grey lines mark the separation of major clades and subclades/species not classified into major clades. The darker the square, the higher the amplification frequency of that marker for the sample group.

**Figure 3:**
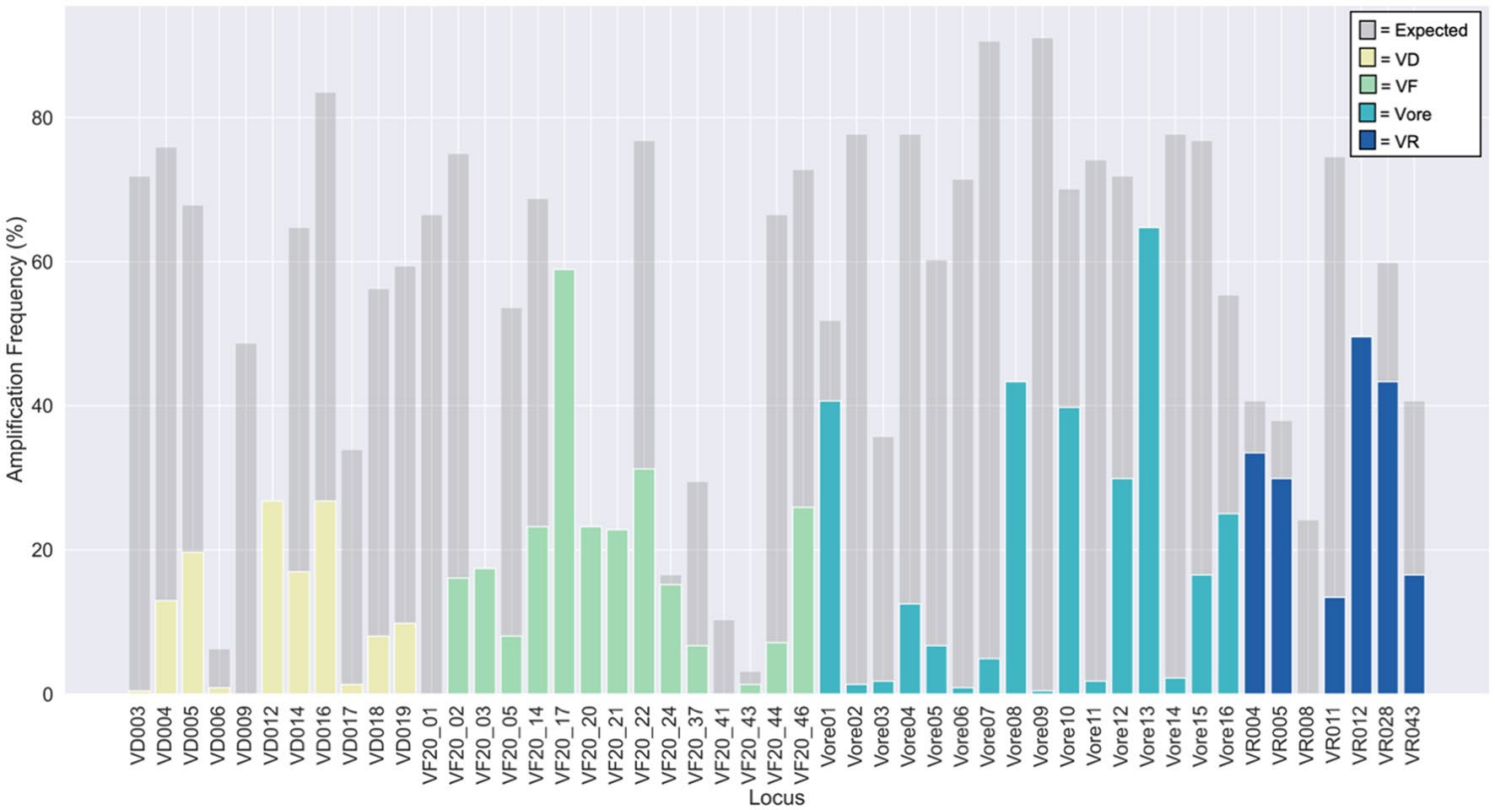
Overall expected and spurious amplification frequency percentages for all 49 SSR markers. Expected amplification is shown in grey. Spurious amplification is shown in color. Yellow represents the *Viburnum dilatatum* (VD) markers, green represents the *V. farreri* (VF) markers, teal represents the *Oreinotinus* subclade (Vore) markers, and blue represents the *V. rufidulum* (VR) markers.

The marker set with the overall highest amplification rate was Vore (*Oreinotinus*; 66%) and the lowest amplification percentage was VF (*V. farreri*; 40%). The VD (*V. dilatatum*; a member of *Succotinus* subclade and of *Laminotinus* major clade) markers amplified the most loci in *Succotinus* (95%), *Coriacea* (*Laminotinus*; 83%), and *Lobata* (*Laminotinus*; 82%; Table 1). The VF (*V. farreri*; *Solenotinus* subclade and *Crenotinus* major clade) markers amplified the most loci in *Solenotinus* (70%), *Lutescentia* (*Crenotinus*; 68%), and *Dentata* (*Porphyrotinus*; 58%). The Vore (*Oreinotinus* subclade; *Porphyrotinus* major clade) markers amplified the most loci in *Succotinus* (*Laminotinus*; 98%), *Dentata* (*Porphyrotinus*; 97%), and *Mollotinus (Porphyrotinus*; 97%). The VR (*V. rufidulum*; *Lentago* subclade and *Valvatotinus* major clade) markers amplified the most loci in *Lentago* (94%), *Euviburnum* (*Valvatotinus*; 70%), and *Dentata* (*Porphyrotinus*; 64%). The frequency of amplification per marker per subclade was also calculated (Fig. 2). The markers developed for the *Oreinotinus* subclade amplified loci in species the most uniformly across all subclades. To aid in the selection of markers for future studies, overall in range/expected and spurious amplifications were calculated for each marker (Fig. 3). More expected amplifications did not always coincide with fewer spurious amplifications.

**Table 1:** Amplification percentages per SSR marker set and subclade.

Clade	Subclade	VD	VF	Vore	VR
		N = 11	N = 15	N = 16	N = 7
		Amp (%)	Amp (%)	Amp (%)	Amp (%)
*Valvatotinus*					
	*Lentago*	60	45	73	94
	*Punctata*	51	36	50	57
	*Euviburnum*	57	35	68	70
*Crenotinus*					
	*Solenotinus*	39	70	62	63
	*Lutescentia*	44	68	74	44
*Laminotinus*					
	*Succotinus*	95	49	98	37
	*Lobata*	82	47	83	41
	*Coriacea*	83	49	74	38
	*Sambucina*	68	36	82	17
*Porphyrotinus*					
	*Mollotinus*	71	48	97	52
	*Dentata*	74	58	97	64
	*Oreinotinus*	48	19	58	11
N/A					
	*Pseudotinus*	45	49	74	43
	*Urceolata*	39	18	23	30
	*Opulus*	73	55	95	63
	*Tinus*	81	40	80	23
	*V. clemensiae*	45	50	38	7
	*Viburnaceae*	8	9	14	17
	*Caprifoliaceae*	9	9	23	26

*VD Viburnum dilatatum* markers; *VF V. farreri* markers; *Vore V. Oreinotinus* subclade markers; *VR V. rufidulum* markers; *N* number of markers in the set; *Amp* percent amplification within the expected base pair size.

### Validation: comprehensive SSR marker set

**Figure 4:**
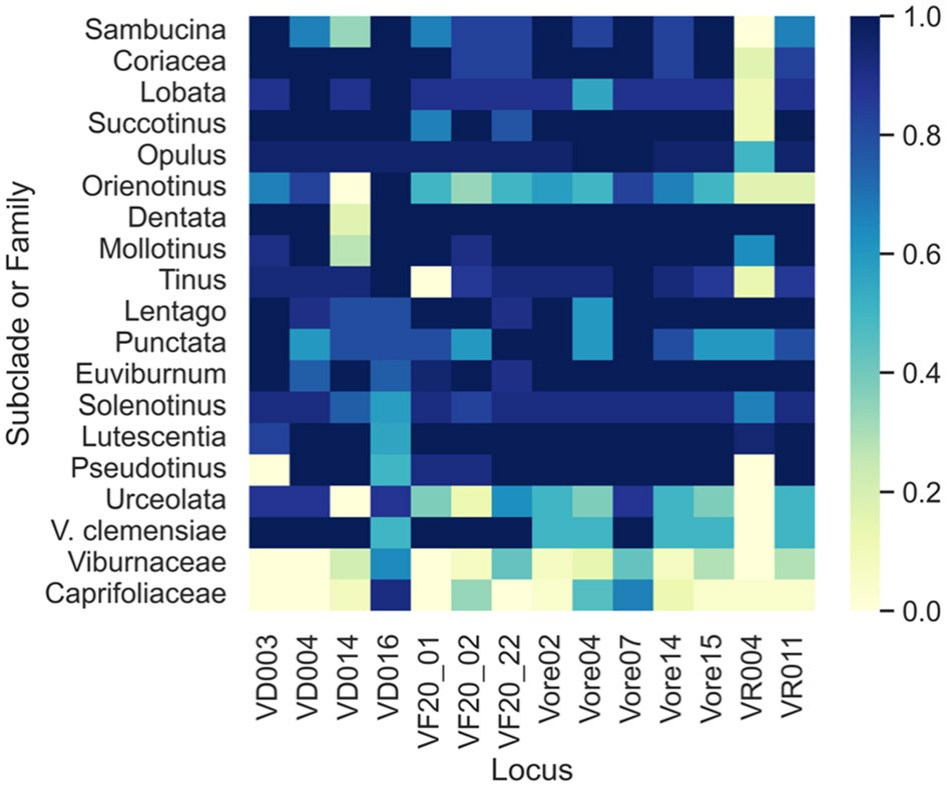
Heatmap of frequency of amplification per subclade or family for the 14 comprehensive SSR markers. The darker the square, the greater the amplification frequency was for the group of samples.

Fourteen markers were selected as a starting point for future research with any *Viburnum* species, regardless of clade. These markers included VD003, VD004, VD014, VD016, VF20_01, VF20_02, VF20_22, Vore02, Vore04, Vore07, Vore14, Vore15, VR004, and VR011, which displayed high amplification rates across most subclades (Fig. 4). This set of markers successfully amplified loci in 74% of all samples, including 85% of *Viburnum* species samples and 19% of species not classified in the *Viburnum* genus. This comprehensive marker set had high amplification frequencies and detected heterozygosity in the four species selected for validation (Table 2). The observed heterozygosity ranged from 0 to 1 with an average of 0.31 in *V. carlesii*, 0.48 in *V. opulus*, 0.35 in *V. plicatum*, and 0.50 in *V. tinus*. The amplification rates and observed heterozygosity were not as high in the species outside of *Viburnum*, from which amplification success was an average of 19% in the rest of the Viburnaceae and 20% in Caprifoliaceae. The average observed heterozygosity was 0.10 for the species of the Viburnaceae and 0.01 for species in the Caprifoliaceae.

**Table 2:** Comprehensive 14 SSR marker set amplification percentages and observed heterozygosity in *Viburnum carlesii* (*Euviburnum* subclade), *V. opulus* (*Opulus* subclade)*, V. plicatum* (*Lutescentia* subclade), and *V. tinus* (*Tinus* subclade).

Locus	*V. carlesii* (*Euviburnum*)		*V. opulus* (*Opulus*)		*V. plicatum* (*Lutescentia*)		*V. tinus* (*Tinus*)		*Viburnaceae*		*Caprifoliaceae*	
	N = 14		N = 18		N = 15		N = 9		N = 14		N = 24	
	Amp (%)	H_o_	Amp (%)	H_o_	Amp (%)	H_o_	Amp (%)	H_o_	Amp (%)	H_o_	Amp (%)	H_o_
VD003	100	0.14	94	0	100	0.6	100	0.56	0	0	0	0
VD004	79	0.09	94	0.82	100	0	100	0.56	0	0	0	0
VD014	100	0.07	94	0.47	100	0.8	100	0.78	21	0	8	0
VD016	100	0.07	94	0	67	0.2	100	0.89	64	0.89	92	0
VF20_01	100	0.14	94	0.35	100	0.4	0	0	0	0	0	0
VF20_02	100	0.50	94	0.12	100	0.47	100	0.78	7	0	33	0
VF20_22	93	0.92	94	0.24	100	1.00	100	0.78	43	0.17	0	0
Vore02	100	0	94	1.00	100	0.27	100	0	7	0	4	0
Vore04	100	0.64	100	0	100	0	100	0.44	14	0	46	0
Vore07	100	1.00	100	1.00	100	0.07	100	1.00	43	0.33	67	0.13
Vore14	100	0	94	0.82	100	0	100	0	7	0	13	0
Vore15	100	0.43	94	0.88	100	0.67	100	0.89	29	0	4	0
VR004	100	0.14	67	0	100	0.47	0	0	0	0	4	0
VR011	100	0.21	94	1.00	100	0	100	0.33	29	0	4	0
Mean	98	0.31	93	0.48	98	0.35	86	0.50	19	0.10	20	0.01

*N* number of individuals sampled; *Amp* percent amplification within the expected base pair size; *Ho* Observed heterozygosity.

## Discussion

### Amplification within expected versus outside size range

The transferability of SSR markers between individuals, species, and genera is dependent upon the conservation of the primer sites. Despite primer sequences being around 20 bp in length, they can bind to locations in the genome other than the target locus, causing spurious banding. This is especially prevalent in cross-amplification studies. Anything greater than 50 bp from the published expected range was considered a different locus than the target one, which happened in markers across all four sets and was not concentrated in a set from a single source. This 50 bp cutoff was selected because the majority of strong peaks with little to no spurious banding occurred within 50 bp of the expected size range. Consequently, some of the amplicons outside of the 50 bp cut-off could be from the target loci. Sequencing the amplicons would resolve this uncertainty, but sequencing was outside the scope of this unusually large study. However, sequencing amplicons is highly recommended in cross-amplification studies due to the potential of homoplasy^33,39^.

Future research with these markers should involve sequencing amplicons from species of distantly related *Viburnum* subclades from the marker source species to confirm the expected locus was amplified. Additionally, amplicons that were greater than 50 bp outside of the expected range should be sequenced to confirm they do not contain the expected SSR. This could be especially helpful for potentially using these markers in the Caprifoliaceae and the other species in the Viburnaceae. Many markers produced clean, strong bands in samples from outside of *Viburnum*, but most amplicons were too far removed from the expected size range to assert they were the correct locus without sequencing. After sequencing, more of the markers could potentially be used for a larger variety of species. Additionally, increasing the annealing temperature in the PCR protocol could help eliminate amplifying non-target loci.

### Overall amplification

The transferability of SSR markers throughout plant species overall is lower than in animals^43^. The cross-amplification success of markers in eudicots is about 71%, with amplification success within genera almost 80% and among genera almost 60%^43^. A study with nine Rosaceae species determined *Prunus* SSR markers had cross-species amplifications of 84% within the genus and 38% outside *Prunus*^41^. Despite the comparable size of the *Prunus* genus, with about 200 species^48^, *Viburnum* markers displayed much lower cross-amplification with 60% within genus and 14% outside of the genus. However, more than five times the species were included in our study.

The overall trends of marker amplification success aligned with the subclades the markers were developed from. VD markers amplified the highest percentage of target loci in the *Laminotinus* and *Porphyrotinus* major clades. VF markers amplified the most in the *Crenotinus* and *Porphyrotinus* major clades. VR markers amplified the most in the *Valvatotinus* and *Crenotinus* major clades*.* Additionally, VR markers amplified the highest percentage of target loci in the Viburnaceae and Caprifoliaceae out of the four marker sets. Vore markers had high amplification success in most clades, which is likely due to the SSRs initially being identified in four different species and the markers subsequently developed in two species^30^. This process was effectively selecting for markers that displayed cross-amplification from the beginning. Despite this, the amplification frequency of Vore markers in the *Oreinotinus* subclade was low compared to most other subclades. The leaf samples for the *Oreinotinus* subclade were from herbaria and collected between 1935 and 1998, therefore the sample age is likely a contributing factor to DNA degradation and the resulting low amplification success. The older sample age also likely explains the relatively low amplification success of all 49 makers in the *Oreinotinus* subclade.

The Vore markers that had particularly low amplification frequency in *Oreinotinus* were not initially discovered in species in the *Oreinotinus* subclade^30^. Vore05 was originally from *V. trilobum* (*Opulus* subclade) and had 0% amplification in the *Oreinotinus* subclade. The Vore16 locus was isolated from *V. dentatum* (*Dentata* subclade) and only had 17% amplification in the *Oreinotinus* subclade. It should be noted Vore09 was mined from mitochondrial NGS data of *V. dentatum* and despite the marker amplifying loci well and being polymorphic, all samples were inherently homozygous at this locus and special consideration should be taken before use^30^.

### Phylogenetic and morphological classification comparisons

The phylogenetic position of lone species (i.e., *V. clemensiae* and *V. amplificatum*) and subclades (i.e., *Pseudotinus,* and *Urceolata*) have been changed as additional data has accrued. *Viburnum clemensiae* has been placed as sister to the rest of *Viburnum*^20,22,24^, but recently has been considered sister to a clade containing *Crenotinus, Valvatotinus,* and subclades *Pseudotinus* and *Urceolata*^1^. VF and VD markers had the highest amplification rate (50% and 45%, respectively) in *V. clemensiae*. Additionally, *V. farreri* and *V. clemensiae* are two of the few species that have panicle-like inflorescence. This could indicate that *V. clemensiae* would be better suited as sister to the clade containing *Crenotinus, Valvatotinus,* and subclades *Pseudotinus* and *Urceolata* as suggested in Landis et al.^1^, rather than as sister to the whole genus.

*Pseudotinus* has been classified as a polytomy with *Valvatotinus* and rest of *Viburnum* (*Pluriviburnum*)^22^, sister to *Valvatotinus*^20^, and sister to *Urceolata*^1,24^. Considering the morphological features of buds, leaf margin, inflorescence architecture, and extrafloral nectaries, this subclade shares the most features with *V. rufidulum* in *Valvatotinus*^22^. However, VR markers displayed the lowest amount of amplification to this subclade. The results of this study therefore do not support the placement of *Pseudotinus* as sister to *Valvatotinus*, and perhaps sister to *Urceolata* is more appropriate.

*Urceolata* has been classified as sister to *Amplicrenotinus* (*Crenotinus* + *V. amplificatum*)^20,22^ and sister to *Pseudotinus*^1,24^. VF (*Crenotinus*) markers exhibited the lowest amplification success in *Urceolata*, which supports not classifying *Urceolata* as sister to *Amplicrenotinus*. *Urceolata* does not share many morphological features with any of the species markers were developed from, but interestingly it shares the least morphological features with *V. dilatatum*^22^. The only feature they share is the umbel-like inflorescence, which is present in most *Viburnum* species. Despite this, VD markers had the highest amplification percentage at 39% across *Urceolata* species.

The taxonomic placement of the *Viburnum* genus is also under debate and whether the family should be called either Adoxaceae or Viburnaceae, or if they should be separate families^3^. The amplification frequencies of the markers in the outgroup species of the Viburnaceae and Caprifoliaceae were low and similar, which perhaps suggests the Viburnaceae and Adoxaceae should be separate families, with *Sambucus* and *Adoxa* in Adoxaceae.

### Validation: comprehensive SSR marker set

The primary goal of this study was to evaluate the cross-amplification success of the 49 developed markers to guide future research and classification in *Viburnum*. Depending on the application, some markers will have higher amplification rates and polymorphisms than others, but as an initial go-to set, the following 14 markers are recommended: VD003, VD004, VD014, VD016, VF20_01, VF20_02, VF20_22, Vore02, Vore04, Vore07, Vore14, Vore15, VR004, and VR011. If investigating a species outside of *Viburnum*, markers not included in this comprehensive set would likely have higher amplification success. Adding markers with higher amplification success outside of *Viburnum* such as VF20_17, VF20_21, Vore10, Vore12, VR028, and VR043 to markers VD014, VD016, VF20_02, VF20_22, Vore04, Vore07, Vore15, and VR011 within the comprehensive set would likely yield better results for studies concerning species not classified in the *Viburnum* genus.

This comprehensive marker set provides coverage of all subclades and the proof-of-concept species. Similar to the overall study, this subset of the dataset in general also demonstrates that the conservation of the primer site is correlated with the evolutionary distance between the species used in marker development and the sample species. *Viburnum carlesii* is a member of the *Euviburnum* subclade and *Valvatotinus* major clade, which helps to explain VD004 (from *Laminotinus* major clade) having the lowest amplification rate. *Viburnum opulus* is a member of the *Opulus* subclade, which is not classified in any of the four major clades but is more closely related to the *Laminotinus* major clade, which possibly explains VR004 having the lowest amplification rate.

Despite these comprehensive SSR markers displaying wide cross-amplification, they still also have relatively high observed heterozygosity rates and thus, are useful for genetic diversity studies of any specific species. The high observed heterozygosity rates demonstrate the markers’ ability to identify different alleles in individuals. To the best of our knowledge, this makes this comprehensive marker set the first marker set capable of characterizing any species in a large genus. This will save time and resources in future research as no additional markers will need to be developed. Once sequencing is performed on select amplicons, these comprehensive SSR markers will be ready for use within *Viburnum*, likely with limited PCR optimization. Future applications for these markers could include characterization and population-level studies for any species within or closely related to *Viburnum* as well as adding them to established linkage maps^49^ for future breeding efforts.

## Supplementary Information

Supplementary Information.

## Data Availability

All data generated or analyzed in this study are included in this published article and its supplementary information files.
